# Application of Automatic Kinematic Analysis Program for the Evaluation of Dysphagia in ALS patients

**DOI:** 10.1038/s41598-019-52246-x

**Published:** 2019-10-30

**Authors:** Ban Hyung Lee, Jun Chang Lee, Sun Myoung Lee, Yulhyun Park, Ju Seok Ryu

**Affiliations:** 1 Department of Rehabilitation Medicine, Seoul National University Hospital, Seoul, South Korea; 2 Department of Rehabilitation Medicine, Seoul National University Bundang Hospital, Seongnam, South Korea; 3 Department of Rehabilitation Medicine Seoul National University Bundang Hospital, Seoul National University College of Medicine, Seongnam, South Korea

**Keywords:** Neurology, Engineering

## Abstract

Dysphagia in amyotrophic lateral sclerosis (ALS) increases the risk of malnutrition, dehydration, and aspiration pneumonia. Kinematic analysis of videofluoroscopic swallowing study (VFSS) can provide detailed movement of the hyoid bone, revealing abnormalities of swallowing in ALS patients. We developed an automated kinematic analysis program (AKAP) that analyzes the trajectory of the hyoid bone via a visual tracking method. The aim of this study was to investigate the hyoid movement in ALS patients using AKAP and compare it with non-dysphagic subjects. Thirty ALS patients who underwent VFSS in Seoul National University Bundang Hospital between 2015 and 2017 were recruited. For comparison, 30 age-matched control subjects were also enrolled; the same swallowing study was conducted using thin fluid and yogurt. The hyoid bone movement was analyzed by evaluating the vertical and horizontal distances with four peak points (A, B, C, D), and the time of each point were also calculated. With respect to distance parameters, only vertical peak distance (distance between B, D points) during thin fluid swallowing was significantly decreased in ALS patients. (p = 0.038) With respect to temporal parameters, Time ABC, Time ABCD, and Duration C were significantly increased in ALS patients when swallowing both thin fluid and yogurt. (Time ABC p = 0.019, p = 0.002; Time ABCD p = 0.001, p = 0.004; Duration C p = 0.004, p = 0.025 respectively). This result revealed that dysphagia in ALS patient is caused by decreased velocity of hyoid bone movement due to the development of weakness in swallowing-related muscles. The parameters of kinematic analysis could be used to quantitatively evaluate dysphagia in motor neuron disease.

## Introduction

Dysphagia is one of the most common clinical symptoms presented in patients with amyotrophic lateral sclerosis (ALS) after disease progression. Although there is bulbar involvement in the late phase of the disease, about one-third of ALS patients complain of swallowing difficulties at the onset^1^.

The degeneration of bulbar motor neurons leads to swallowing dysfunction, decreased movement, and fasciculation of the tongue^2^. The oral and pharyngeal phases of swallowing are most vulnerable to aspiration due to the degeneration of the motor nuclei of lower cranial nerve in ALS patients^3^. After the diagnosis of dysphagia, ALS patients require enteral tube feeding through a percutaneous endoscopic gastrostomy (PEG)^4^ or nasogastric tube to minimize the risk of aspiration pneumonia and malnutrition^5,6^.

There are several tools to evaluate dysphagia, including videofluoroscopic swallowing study (VFSS), fiberoptic endoscopic evaluation of swallowing (FEES), and oro-pharyngeal esophageal scintigraphy (OPES). Among these, VFSS is considered to be the primary tool for evaluating oropharyngeal dysphagia^7^.

The abnormalities found during the pharyngeal stage cause direct effects on penetration or aspiration. The pharyngeal stage begins when the hyoid bone begins to move in the anterior-superior direction by contractions of digastric, geniohyoid, and mylohyoid muscles. Then, the hyoid bone moves back to its neutral position by the contractions of sternohyoid, omohyoid, and thyrohyoid muscles^8,9^. Movement of the hyoid bone is related to the elevation of the larynx and opening of the upper esophageal sphincter^9,10^. Therefore, the kinematic analysis of hyoid bone movement is essential in the evaluation of swallowing function.

The kinematic analysis of VFSS, which can detect subtle abnormalities, provides quantitative information about the bolus and hyoid movement in swallowing^9,11–13^. However, the manual kinematic analysis is not applicable in practical cases, since it requires a lot of time and effort. Moreover, the result could vary depending on the skill level of the examiner. In order to provide accurate kinematic analysis of VFSS quickly, we used an automated kinematic analysis program (AKAP, C-2015-019815) that analyzed the trajectory of the hyoid bone via a visual tracking method^14^.

There have been some studies analyzing the kinematic movement of the hyoid bone using VFSS. Previous studies revealed a significant difference in the duration and extent of hyoid bone movement by age^15–17^, food bolus volume^10^, and food characteristics^11,12,18^. Previous studies also revealed that the hyoid movement is reduced in patients with myopathy and muscular dystrophy, compared with their healthy counterparts^16,19^. Thus far, however, there has not been a study using kinematic analysis of hyoid bone movement in ALS patients. In a previous study using high resolution manometry, pharyngeal weakness was significantly correlated with the development of dysphagia and the severity of dysphagia^7^. Given that the movement of the hyoid bone relies on contractions of the supra, infra, and retrohyoid muscles, hyoid bone movement may also be impaired in ALS patients. Therefore, we speculated that a kinematic analysis may reveal characteristic abnormal findings in ALS patients compared with non-dysphagic subjects. The purpose of this study was to evaluate the differences in hyoid motions between ALS patients and normal subjects. We hypothesized that the vertical and horizontal extent, as well as the duration of hyoid movements in ALS patients may be different from those in normal subjects. Findings presented in this study may reveal the cause of dysphagia in MND patients and establish a relevant strategy for swallowing rehabilitation.

## Methods

### Participants

Thirty ALS patients with swallowing disorders who have previously undergone VFSS study at Seoul National University Bundang Hospital were retrospectively recruited between 2015 and 2017. All VFSS video clips were stored. The average age of those in the study group was 67.63 ± 12.14 years; 12 males and 18 females. For comparison, 30 age-matched control subjects without swallowing or gastrointestinal disorders who have previously undergone VFSS at the same hospital were retrospectively recruited. The average age of the control group was 64.87 ± 14.54 years; 14 males and 16 females. The study group was diagnosed according to the revised El Escorial criteria. All of these participants were examined by VFSS with thin fluid and yogurt.

The study was conducted in accordance with the Declaration of Helsinki for the participation of human subjects in research. This study was approved by the institutional review board (IRB) of Seoul National University Bundang Hospital (IRB no. B-1506-304-110), and the requirement for informed consent was waived.

### Videofluoroscopic swallowing study procedures

All included subjects were given 3 ml of 35% W/V diluted barium solution as thin fluid (IDDSI Level 0) and 5cc of Yoplait with 30% W/V diluted barium solution (IDDSI Level 3) as yogurt. Both of these were taken using a plastic spoon. For better visualization of soft tissues of the pharyngeal structures, an X-ray beam was set to 40 kV peak. The study and control groups underwent the same VFSS procedure. For all participants, the severity of dysphagia was expressed on Videofluoroscopic Dysphagia Scale (VDS), and the degree of aspiration was expressed on Penetration-Aspiration Scale (PAS). All videos were stored on the hard disk.

### Kinematic analysis of hyoid bone movement

VFSS was recorded from the lateral view with a 30-per-second video frame rate. We used two coins with a diameter of 2.4 cm as the position index and scale for kinematic analysis. The coins were taped under the subjects’ chin and on the lateral side of the face, where the mastoid process was prominent.

For the kinematic analysis of hyoid bone movement, Automated Kinematic Analysis Program (AKAP) was used (Software Registration Number: C-2015-019815). VFSS video clips, which were recorded as an AVI file format, were divided into serial frame images and sequentially displayed on UI screen by AKAP using Matlab. When the automatic hyoid bone tracking program was activated, the first frame of VFSS video clip popped up on the screen, then the examiner was asked to mark the reference points in the following manner: anterior border of the hyoid bone, center of the coin at mandible, anteroinferior edge of the C3 vertebral body, and C4 anterosuperior edge of the C4 vertebral body. Then Matlab automatically marked the remaining frames and analyzed hyoid bone movement based on the coin, C3, and C4 vertebral bodies^14^.

We defined V3(n)/V4(n) as the anteroinferior point of C3/C4 in n^th^ frame. The Y-axis was defined as the line between V3(1) and V4(1). The X-axis was derived as the line perpendicular to the Y axis. The coordination of each frame was matched to increase accuracy. The V3(n)-V4(n) vector was extracted from each n^th^ frame. This V3(n)-V4(n) vector was rotated and moved to be in the same direction and position as the V3(1)-V4(1) reference vector. Within these matched frames, the trajectory of the point that we marked on the anterior border of hyoid bone was drawn.

**Figure 1:**
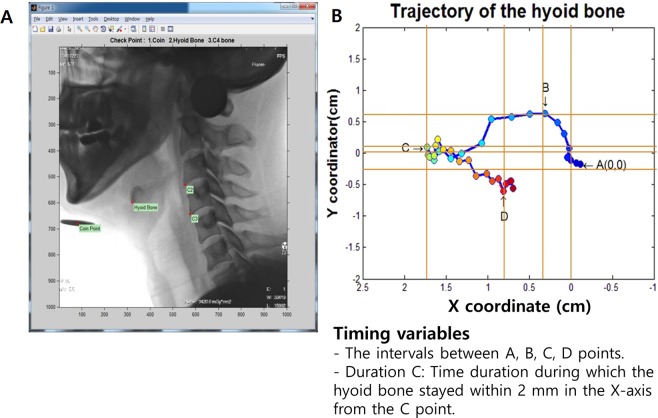
This figure shows the automated kinematic analysis program (AKAP, C-2015-019815). After marking a point on the anterior border of the hyoid bone, Antero-inferior edge of the C3 vertebral body and C4 Antero-superior edge of the C4 vertebral body of the first frame, then, the MATLAB automatically analyzed the position of the hyoid bone based on C3 and C4 vertebral bodies of all last frames, respectively. (**B**) AKAP measures the timing and distance parameters between (**A**–**D**) points and the duration which the distance maintained at 2 mm or less from point C.

We analyzed the temporal and distance parameters of the hyoid bone’s trajectory. In the kinematic movement of the hyoid bone in normal subjects, the hyoid bone initially moved anteriorly and superiorly, and then inferiorly and posteriorly. Point A was marked as the initial position of the hyoid bone. Point B was marked as the maximal superior position of hyoid bone. Then, the hyoid bone moved anteriorly as it arrived at the maximal anterior position, which was defined as point C. Finally, point D was marked as the maximal inferior position of the hyoid bone (Fig. 1). We measured and calculated the temporal and distance parameters between each point from A-D. Moreover, we also measured the duration of time in which the hyoid bone remained within 2 mm or less from point C (duration of C state)^14^.

### The accuracy of the automated kinematic analysis program (AKAP)

There was a previous study to evaluate the accuracy of the Automated Kinematic Analysis Program (AKAP). Randomly selected 50 swallowing VFSS video clips from 8 dysphagic patients with various etiologies and 19 video clips from 10 healthy subjects were utilized for performance verification. These video clips contained both non-mandibular overlapping and mandibular overlapping with hyoid bone during swallowing. In the result, percent errors of the hyoid bone tracking was 1.7 ± 2.1% for non-mandibular overlapped video clips and 4.2 ± 4.8% for overlapped video clips. In a comparison between manually performed and automatically performed moving trajectories of the hyoid bone, the correlation coefficients were 0.986 ± 0.017 (X-axis) and 0.992 ± 0.006 (Y-axis) for non-overlapped video clips, and 0.988 ± 0.009 (X-axis) and 0.991 ± 0.006 (Y-axis) for overlapped video clips. Based on this previous study, AKAP showed high correlation with manual extractions while reducing intra- and inter-examiner variability^14^.

### Statistical analysis

Data analyses were performed using Statistical Package for the Social Sciences. (SPSS 19; SPSS Inc. Chicago, Illinois) The mean and standard deviation were derived for the study and control groups, and independent t-test and chi-square test were used to make a comparison between the two groups.

To analyze the association between time parameter of AKAP variables and the duration of disease, a linear regression analysis was performed, controlling for age and gender as confounding factors. In all analyses, p-values of less than 0.05 were considered statistically significant.

## Results

### Demographic data of ALS patients and control subjects

The average age and gender were not significantly different between the study and control groups (p = 0.417; p = 0.461 respectively). The average onset of ALS to VFSS examination date was 17.86 ± 13.34 months. The proportion of ALS type diagnosed by revised El Escorial criteria were as follows: 11 possible ALS (36.7); 11 probable ALS (36.7); 8 definite ALS (26.7).

### Comparisons of VFSS and AKAP parameter between ALS patients and control subjects

In the study group, the mean VDS, PAS (thin fluid), and PAS (yogurt) were 29.08 (±21.52), 2.63 (±2.03), and 1.57 (±1.46), respectively. On the other hand, the swallowing function was normal in the control group; therefore, the VDS score was 0 and PAS score was 1. (VDS: lowest score 0, PAS: no aspiration score 1).

AKAP parameters were divided into either a distance or time parameter. Among the distance parameters of AKAP, the vertical distance (BD) in the study group was significantly increased than in the control group (p = 0.038) during thin fluid swallowing. However, other distance parameters (i.e. vertical AB, vertical AC, vertical AD, horizontal AB, horizontal AC, and horizontal AD) were not significantly different between the two groups, for both thin fluid and yogurt (p > 0.05).

Among the timing variables of AKAP for the thin fluid, Time ABC, Time ABCD, and Duration C were significantly delayed in the study group (Time ABC: p = 0.019, Time ABCD: p = 0.001, Duration C: p = 0.004). Similarly, among the timing parameters for swallowing yogurt, Time ABC, Time ABCD, and Duration C were significantly delayed in the study group (Time ABC: p = 0.002, Time ABCD: p = 0.004, Duration C: p = 0.025) [Table 1].

**Table 1:** Comparisons of VFSS and AKAP parameter between ALS patients and control subjects.

VFSS variables	ALS patients (n = 30)		Control (n = 30)		P value	
VDS	29.08 (±21.52)		0 (±0)		**<0.001**	
PAS (fluid)	2.63 (±2.03)		1 (±0)		<0.001*	
PAS (Yogurt)	1.57 (±1.46)		1 (±0)		0.048*	
**AKAP parameters**	**Fluid**	**Yogurt**	**Fluid**	**Yogurt**	**Fluid**	**Yogurt**
Vertical AB (cm)	1.22 (0.67)	1.63 (1.13)	1.15 (0.66)	1.17 (0.70)	0.709	0.062
Verical AC	0.64 (0.98)	1.13 (1.13)	0.83 (0.76)	0.89 (0.89)	0.384	0.36
Vertical BD	1.70 (0.73)	1.87 (1.26)	1.37 (0.43)	1.43 (0.70)	**0.038**	0.098
Vertical AD	−0.48 (0.8)	−0.24 (0.86)	−0.21 (0.70)	−0.34 (0.92)	0.174	0.644
Horizontal AB	0.79 (0.69)	0.70 (0.61)	0.72 (0.51)	0.89 (0.59)	0.627	0.217
Horizontal AC	1.37 (0.73)	1.36 (0.68)	1.21 (0.42)	1.37 (0.50)	0.313	0.946
Horizontal AD	0.69 (0.67)	0.52 (0.66)	0.50 (0.57)	0.43 (0.53)	0.242	0.548
Time AB (sec)	0.64 (0.53)	0.66 (0.34)	0.52 (0.22)	0.54 (0.24)	0.167	0.119
Time ABC	0.94 (0.46)	0.95 (0.40)	0.71 (0.20)	0.70 (0.14)	**0.019**	**0.002**
Time ABCD	1.62 (0.59)	1.60 (0.71)	1.18 (0.33)	1.17 (0.28)	**0.001**	**0.004**
Duration C (sec)	0.36 (0.17)	0.27 (0.13)	0.25 (0.09)	0.21 (0.07)	**0.004**	**0.025**

Values represent means ± SD; Analysis of t-test, equal variances assumed (Same results in equal variances not assumed).

*χ^2^ test.

AKAP: Automated Kinematic Analysis Program, VDS: Videofluoroscopic Dysphagia Scale, PAS: Penetration-Aspiration Scale.

### The association between time parameter and time interval of ALS diagnosis to VFSS examination

**Figure 2:**
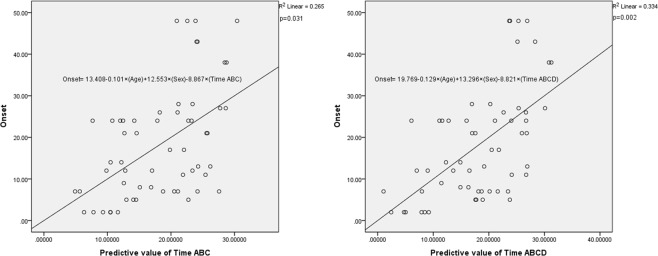
Association (**a**) between predictive value of Time ABC with confounding factors (age and sex) and onset (time interval of ALS diagnosis to VFSS examination), and (**b**) between Time ABCD with confounding factors (age and sex) and onset (time interval of ALS diagnosis to VFSS examination). (*Age = years; Sex = male: 1, female: 2).

According to the linear logistic regression analysis, Time ABC and Time ABCD were negatively associated with time interval of ALS diagnosis to VFSS examination. However, Duration C was not associated with the time interval (ß = −0.263, p = 0.031; ß = −0.374, p = 0.002; ß = 0.063, p = 0.613, respectively) [Table 2] [Fig. 2].

**Table 2:** The association between time parameter and time interval of ALS diagnosis to VFSS examination.

Variables		ALS patient (p value)
Time ABC	Adjusted R2	0.265
	ß	−0.263
	p	0.031
Time ABCD	Adjusted R2	0.334
	ß	−0.374
	p	0.002
Duration C	Adjusted R2	0.202
	ß	0.063
	p	0.613

Linear logistic regression analysis; Dependent variables: time interval of ALS diagnosis to VFSS examination; Independent variables: age, gender.

## Discussion

In this study, we found that the timing parameters were significantly different between ALS patients and aged-matched non-dysphagic subjects. The parameters, Time ABC, Time ABCD, and Duration C were significantly delayed in patients with ALS, except for the Time AB. This finding indicates that the hyoid bone movement is delayed after reaching point B. In other words, the velocity of hyoid bone appears to decrease after reaching the vertical peak. Although there have been some studies evaluating hyoid bone movement with respect to age and other etiologies, the results have been inconsistent. Kendall *et al*. (2001) reported delayed hyoid movement in elderly people without an obvious medical or surgical cause for dysphagia; however, Kang, B. S. *et al*. (2010) reported no significant findings in the temporal measurement of hyoid bone movement^15,20^. In previous studies evaluating other etiologies, duration of hyoid bone was delayed only in patients with myopathy, but not in those with myotonic muscular dystrophy or stroke^16,19^. If hyoid bone motion is slowed in elderly people without neurologic diseases and myopathic patients as previously suggested, then hyoid motion may be related to the development and severity of dysphagia.

In a previous study, it was shown that distal degeneration may occur in longer fast-fatigable α-motor axons innervating type IIb muscle fibers before axons innervating type I muscle fibers in muscles with mixed fiber types^21^. It is also known that both immunofluorescence and RT-PCR demonstrated a selective decrease in the expression levels of the genes encoding the myosin heavy chains specific to fast-twitch fibers in SBMA subjects^22^. These findings suggest that the degeneration in fast-fatigable α-motor axons innervating type IIb muscle fibers in ALS patients contributed to a slower velocity of hyoid bone movement and the development of dysphagia^23,24^.

Regarding distance parameters, our study showed that only one distance parameter, the maximal vertical distance of hyoid movement (vertical BD), was significantly increased in ALS patients when swallowing thin fluid. Previous studies yielded inconsistent results with respect to distance parameters of hyoid bone movement. Some studies reported an increased extent of hyoid displacement as a compensatory mechanism^15,20^. However, myopathy patients showed a decreased extent of both vertical and horizontal hyoid bone movements due to weakness in all hyoid related muscles. Our results support previous findings in that vertical hyoid bone movement is increased as a compensatory mechanism; however, the increased motion is minimal due to muscular weakness resulting in decreased hyoid bone movement.

To the best of our knowledge, there has not been a study analyzing the movement of hyoid bone kinematically in ALS patients. Our study is noteworthy in that we applied a kinematic analysis for the first time, and AKAP contributed to the understanding of the pathophysiology of dysphagia in ALS patients.

In the present study, unlike myopathy patients, ALS patients showed an increased extent of vertical hyoid bone movement and normal duration of hyoid elevation (Time AB). Moreover, the longer time interval from ALS diagnosis to VFSS examination also suggests an association with the delay in Time ABC and Time ABCD. This result illustrates the progression of weakness in infrahyoid muscles. These findings suggest a relatively spared function of suprahyoid muscles in ALS patients. In other words, weakness in the infrahyoid muscles, which acts to descend the hyoid bone, develops earlier than in the suprahyoid muscles. This suggests that the pathophysiology of dysphagia in ALS patients may be related to the sequential or selective weakness developed throughout the disease progression.

This study has some limitations. First, the sample size of ALS patients and control subjects was relatively small, with only 30 individuals in each group. However, considering the rarity of ALS disease, it is difficult to have a higher sample size; 30 patients are very valuable and might be enough to be significant. Second, this study analyzed VFSS only with two diets (i.e. thin fluid and yogurt). Study with various other textures and viscosities might also be necessary in the future.

Despite these limitations, the present study makes a valuable contribution to the field, considering that it is the first study evaluating the relationship between hyoid bone movement and dysphagia in ALS patients. Moreover, we used the AKAP for precise analysis. For further research, EMG analysis of the supra and infra hyoid bone muscles in ALS patients would be helpful to better evaluate the weakness of infrahyoid muscles.

In conclusion, dysphagia in ALS patients is caused by decreased velocity of hyoid bone movement due to the development of weakness in swallowing-related muscles, especially in the infrahyoid muscles. Here, parameters of kinematic analysis were used to quantitatively evaluate dysphagia in ALS patients. The disordered mechanism found in this study provides a better understanding for the management of ALS patients with dysphagia.
